# Topical therapy with high-volume budesonide nasal irrigations in difficult-to-treat chronic rhinosinusitis^☆^

**DOI:** 10.1016/j.bjorl.2015.03.014

**Published:** 2015-09-07

**Authors:** Eduardo Macoto Kosugi, Guilherme Figner Moussalem, Juliana Caminha Simões, Rafael de Paula e Silva Felici de Souza, Vitor Guo Chen, Paulo Saraceni Neto, José Arruda Mendes Neto

**Affiliations:** Sector of Rhinology, Department of Otorhinolaryngology and Head and Neck Surgery, Escola Paulista de Medicina, Universidade Federal de São Paulo (EPM/UNIFESP), São Paulo, SP, Brazil

**Keywords:** Sinusitis, Nasal polyps, Therapeutic irrigations, Corticosteroids, Endoscopy, Sinusite, Pólipos nasais, Irrigação terapêutica, Corticosteroides, Endoscopia

## Abstract

**Introduction:**

Chronic rhinosinusitis (CRS) is termed difficult-to-treat when patients do not reach acceptable level of control despite adequate surgery, intranasal corticosteroid treatment and up to 2 short courses of systemic antibiotics or corticosteroids in the preceding year. Recently, high-volume corticosteroid nasal irrigations have been recommended for CRS treatment.

**Objective:**

To assess high-volume budesonide nasal irrigations for difficult-to-treat CRS.

**Methods:**

Prospective uncontrolled intervention trial. Participants were assessed before- and 3 months after nasal irrigation with 1 mg of budesonide in 500 mL of saline solution daily for 2 days. Subjective (satisfactory clinical improvement) and objective (SNOT-22 questionnaire and Lund–Kennedy endoscopic scores) assessments were performed.

**Results:**

Sixteen patients were included, and 13 (81.3%) described satisfactory clinical improvement. SNOT-22 mean scores (50.2–29.6; *p* = 0.006) and Lund–Kennedy mean scores (8.8–5.1; *p* = 0.01) improved significantly. Individually, 75% of patients improved SNOT-22 scores, and 75% improved Lund–Kennedy scores after high volume budesonide nasal irrigations.

**Conclusion:**

High-volume corticosteroid nasal irrigations are a good option in difficult-to-treat CRS control of disease, reaching 81.3% success control and significant improvement of SNOT-22 and Lund–Kennedy scores.

## Resumo

**Introdução:**

A rinossinusite crônica (RSC) de difícil tratamento é aquela inadequadamente controlada com cirurgia, corticosteroides tópicos em *spray* e até dois ciclos de medicação sistêmica em um ano. Atualmente, tem sido preconizado o uso de irrigações nasais de corticosteroides em alto volume para seu tratamento.

**Objetivo:**

Avaliar o uso da terapia tópica de irrigações nasais com budesonida em alto volume nos pacientes com RSC de difícil tratamento.

**Método:**

Estudo prospectivo de intervenção não controlado em RSC de difícil tratamento com 3 meses de terapia tópica de irrigação (1 mg de budesonida diluído em 500 mL de soro fisiológico para ser utilizado em dois dias). Realizada avaliação subjetiva (melhora clínica satisfatória) e objetiva (questionário SNOT-22 e classificação endoscópica de Lund-Kennedy).

**Resultados:**

Foram incluídos 16 pacientes, sendo que 13 (81,3%) consideraram sua melhora clínica satisfatória. Houve melhora significante das médias de SNOT-22 (50,2 a 29,6; p = 0,006) e de Lund-Kennedy (8,8 a 5,1; p = 0,01). Individualmente, 75% dos pacientes apresentaram melhora do SNOT-22 e 75%, do Lund-Kennedy.

**Conclusão:**

A terapia tópica de irrigação de alto volume de corticosteroide é uma boa opção no controle clínico dos pacientes com rinossinusite crônica de difícil tratamento, com controle adequado de 81,3% destes pacientes e melhora significante do SNOT-22 e do Lund-Kennedy.

## Introduction

Chronic rhinosinusitis (CRS) is defined as a chronic inflammatory process of nasal mucosa and paranasal sinuses, lasting more than 12 weeks, without complete resolution of symptoms.^1^ There is a growing perception that CSR is not a single disease, but a spectrum of different diseases with similar clinical presentations, whose common pathophysiological mechanism is a chronic inflammation.^2^ In other words, the term CSR is nothing more than a large label or umbrella that hosts a number of different diseases featuring typically nasal obstruction, rhinorrhea, olfactory changes and/or facial pain.

Although not a life-threatening entity, CSR can be considered potentially serious, taking into account the impact produced in quality of life of affected patients, measured by generic quality of life questionnaires, such as SF-36^3^ or questionnaires specific to the disease, such as Sino-Nasal Outcomes Test (SNOT)-22,^4^ even after treatment.^5^ This great impact becomes even more relevant if we consider that 5.51% of the population over 12 years in São Paulo city meet epidemiological diagnostic criteria for CRS, which corresponds to approximately 500,000 individuals with CRS.^6^

In general, CRS is initially treated medically, followed by surgical treatment if necessary, and supplemented by post-operative topical treatment. The initial medical treatment is also known as “maximal clinical treatment”, because it consists of a combination of different classes of drugs in order to optimize therapy and to avoid surgery. But there is no consensus on the composition and duration of maximal clinical treatment,^7^ with highly variable success rates.^8^, ^9^ In addition, the optimized and prolonged use of drugs such as oral antibiotics and corticosteroids can lead to significant adverse effects; thus, there is an effort for replacing systemic therapy by topical nasal therapy to achieve control of CRS.^10^ The direct administration of the drug to inflamed tissue allows an increased local concentration with less systemic absorption, enhancing therapeutic efficacy.^11^ For this reason, surgery has been considered an essential step in treating CRS, by opening spaces and allow for an adequate distribution of the drug through nasal cavities.^10^, ^12^, ^13^

The objective of CRS treatment is to achieve and maintain control of the disease, which is defined as a state in which patients have no symptoms (or their symptoms do not bother them), combined with nasal endoscopy showing healthy or almost healthy mucosa, and in need only of topical nasal medication.^1^ Due to the large heterogeneity of CRS, there exists a profile of patients who, despite clinical and surgical treatments, continue to experience exacerbation of symptoms and inadequate clinical control. The European Position Paper on Rhinosinusitis and Nasal Polyps (EPOS) 2012 defines difficult-to-treat rhinosinusitis as that entity in which patients do not show an appropriate clinical control level despite sinonasal surgery, intranasal corticosteroids and up to two cycles of antibiotics or systemic corticosteroids in the last year.^1^

Recently there has been a perception that sprays and aerosols are not able to reach paranasal sinuses, and that, in most cases, these products do not even reach the middle meatus area. If that is so, such methods should be disregarded in favor of high-volume methods,^14^, ^15^ with a daily irrigation of at least 200 mL.^11^ In view of evidence of superiority of high volume/pressure solutions with respect to sinus penetration, nasal irrigation with corticosteroids at daily doses ranging from 250 μg to 1 mg of budesonide have been used, with encouraging results.^16^ In this line, subgroups traditionally more difficult to control (e.g., CRS with polyps and increased tissue eosinophilia) had even better therapeutic response than the other subgroups.^10^

Taking into account the promising results of the use of nasal irrigation with corticosteroids, especially in those more difficult cases, the aim of this study was to evaluate the use of topical therapy with high-volume nasal irrigation with budesonide solution in patients with difficult-to-treat chronic rhinosinusitis.

## Method

### Study population

The study included sixteen patients diagnosed with CRS, with or without nasal polyps (CRSwNP or CRSw/oNP, respectively) who met the criteria for difficult-to-treat CRS, i.e. inadequate clinical control after endoscopic sinonasal surgery, use of topical nasal corticosteroids in spray and with up to two cycles of oral antibiotics and/or corticosteroids in the last year, with follow-up in a rhinology outpatient clinic.

The diagnosis of CRS was defined according to search criteria suggested by EPOS 2012.^1^

Patients younger than 18 years or who did not wish to participate in the study were excluded. The study was approved by the Ethics Committee of the Institution under number 940.101.

### Study design

This is a prospective, interventional, uncontrolled study in patients with difficult-to-control CRS. The study intervention was the high-volume topical therapy by nasal irrigation with budesonide solution.

Budesonide was prepared by a manipulation pharmacy (VIP Parma, São Paulo, Brazil) as follows: 200 mL of maximally ground budesonide in a mortar, to which 1 mL of glycerin was added in order to completely dissolve the product. After checking the solubility, this product was transferred to a cup and supplemented with glycerol to make up 20 mL, resulting in a glycerol solution of 1% budesonide. Therefore, each 1 mL contained 10 mg of budesonide; or each 2 drops contained 1 mg of the drug.

Patients were instructed to dilute 2 drops of glycerin solution of budesonide 1% (corresponding to 1 mg of budesonide) in 500 mL of saline solution. Patients were also instructed to divide this 500 mL volume in half, i.e. instilling 250 mL each day for two days, irrigating his/her nostrils with a 20-mL syringe in pulses or jets, and to continue this regimen for 3 months. No previous recommendation was proposed as to frequency of irrigations; the patient should only apply the amount of 250 mL on a daily basis (corresponding to the use of 500 μg/day of budesonide).

Patients were evaluated before and after 3 months of topical irrigation therapy. The following epidemiological characteristics were evaluated: gender, age, type of CSR, presence of comorbidity and number of previous sinonasal surgeries. Moreover, the prescription and use of antibiotics and/or systemic corticosteroids, or of other medications that might interfere with the treatment of CRS during topical therapy by irrigation, was documented.

The subjective outcomes evaluated were subjective improvement and degree of satisfaction post-topical therapy by irrigation. When the topical therapy by irrigation came at an end, patients were asked if there was improvement of their clinical condition (total improvement, partial improvement, no improvement, worsening), and whether they were satisfied with the degree of this subjective improvement (satisfied or dissatisfied). “Therapeutic success” was defined as a satisfactory subjective improvement presented by the patient.

Objective outcomes evaluated were scores of the SNOT-22 questionnaire in Portuguese^4^ and endoscopic classification of Lund–Kennedy.^17^ These outcomes were quantitatively and qualitatively evaluated. Quantitative evaluation of objective outcomes involved statistical calculations comparing pre- and post-topical therapy by irrigation. For the qualitative evaluation, the following parameters were used: the minimally important difference of SNOT-22 = 14 points^4^; therefore, differences in post-pre scores between −7 and +7 were considered as “no improvement”. When pre-post difference scores reached values < −7, they were considered as “improvement”. In the other hand, pre-post differences > 7 were considered as “worsening.”

Absolute scores of SNOT-22 (Portuguese version) between 0 and 8 are considered normal^18^; thus, when the patient showed a difference value < −7 and scores for post-topical therapy by irrigation ≤ 8, were considered as “complete improvement”. As to Lund–Kennedy scores, post-pre differences = zero were considered as “no improvement”, any negative value in the post-pre difference was regarded as “improvement” and any positive value of the difference as “worsening”. A post-topical therapy Lund–Kennedy score = zero was considered “complete improvement”.

Epidemiological characteristics and outcomes were compared among patients with and without satisfactory improvement. Quantitative variables were compared by Student's *t* test or Mann–Whitney *U* test, depending on the homogeneity and normality of samples; and qualitative variables were evaluated by Fisher's chi-squared or Fisher's exact test. For all statistical tests, *p*-values < 0.05 were considered as significant.

## Results

The characteristics of 16 patients with difficult-to treat CRS included in this study are detailed in Table 1. The epidemiological characteristics of the study population are presented in Table 2.

**Table 1:** Patient characteristics and satisfactory subjective improvement.

Patient	Gender	Age	Diagnosis	Asthma	Intolerance to ASA	Rhinitis	Commorbities	Previous surgery	Main complaint	Subjective improvement	Satisfaction
1	F	52	CRSwNP	Yes	No	No	Woakes	5	Post. Rhin.	Partial	No
2	F	61	CRSwNP	No	No	Yes	Hypothyroidism	1	Post. Rhin.	Total	Yes
3	M	50	CRSwNP	Yes	No	Yes	Without	1	Post. Rhin.	Partial	Yes
4	M	60	CRSwNP	No	No	Yes	Without	1	Obstruction	Total	Yes
5	F	29	CRSwNP	Yes	No	No	Without	3	Post. Rhin.	Partial	Yes
6	F	56	CRSw/oNP	No	No	No	Churg–Strauss	2	Obstruction	Total	Yes
7	F	59	CRSwNP	Yes	No	No	Without	1	Ant. Rhin.	Total	Yes
8	F	37	CRSw/oNP	No	No	No	VCID	1	Ant. Rhin.	Partial	Yes
9	M	47	CRSwNP	No	No	No	Intol. Dipirone	1	Hiposmia	Partial	Yes
10	M	64	CRSwNP	No	No	No	Renal Tx	1	Ant. Rhin.	Partial	No
11	F	58	CRSwNP	Yes	Yes	No	Without	1	Ant./Post. Rhin.	Partial	Yes
12	F	53	CRSwNP	Yes	Yes	No	Without	2	Ant. Rhin.	Partial	Yes
13	F	47	CRSw/oNP	No	No	No	Without	2	Post. Rhin.	Total	Yes
14	F	62	CRSw/oNP	No	No	No	Without	2	Obstruction	Without improvement	No
15	F	40	CRSwNP	Yes	No	No	Without	2	Ant. Rhin.	Partial	Yes
16	F	39	CRSw/oNP	No	No	No	Renal Tx	2	Post. Rhin.	Total	Yes

SNOT-22, SinoNasal Outcome Test-22; F, female; M, male; CRSwNP, chronic rhinosinusitis with polyps; CRSw/oNP, chronic rhinosinusitis without polyps; Rhin., rhinorrhea; Ant., anterior; Post., posterior; VCID, common variable immunodeficiency; Tx, transplant.

**Table 2:** Epidemiological characteristics and subjective outcomes.

Data		Groups				
		With satisfactory improvement	Without satisfactory improvement	Total	Test	*p*-value
Patients, total	*n* (%)	13 (81.3)	3 (18.7)	16 (100)		
Female gender	*n* (%)	10 (76.9)	2 (66.6)	12 (75)	Fisher	1
Age (years)	Mean (SD)	48.9 (10.2)	59.3 (6.4)	50.9 (10.3)	T	0.11
Nasosinusal polyposis	*n* (%)	9 (69.2)	2 (66.6)	11 (68.8)	Fisher	1
Asthma	*n* (%)	6 (46.2)	1 (33.3)	7 (53.8)	Fisher	1
ASA intolerance	*n* (%)	2 (15.4)	0 (0.0)	2 (12.5)	Fisher	1
Rhinitis	*n* (%)	3 (18.8)	0 (0.0)	3 (18.8)	Fisher	1
Previous surgeries	Mean (SD)	1.5 (0.6)	2.7 (2.1)	1.8 (1.1)	T	0.09

*n*, number; %, percentage; SD, standard deviation; ASA, acetylsalicylic acid.

Patient #6 (a woman) used 30 mg of prednisone continuously due to a diagnosis of Churg–Strauss. Patient #10 (a male renal transplant patient) used 5 mg of prednisone continuously (in addition to immunosuppressive agents as sirolimus, mycophenolate sodium and tacrolimus), and patient #12 (a woman) used omalizumab 600 mg every 2 weeks due to severe asthma. In face of the severity of comorbidities, the drugs used by these patients were not discontinued during the period of topical therapy by irrigation. The doses of the drugs mentioned did not change during the study, and were similar to the doses used in the six months prior to the study.

After topical therapy by irrigation, we asked questions on subjective outcomes to all 16 patients (detailed in Table 1). Of these, 6 reported overall subjective improvement; 9 informed partial subjective improvement; and only one patient felt no improvement, totaling 15 patients (93.8%) with clinical subjective improvement. In addition to the patient without improvement, two patients with partial subjective improvement were not satisfied with the outcome of their topical therapy by irrigation, totaling 13 patients (81.3%) with satisfactory subjective improvement. All patients who did not exhibit satisfactory subjective improvement received an indication for a new surgical procedure, with orientation to restart their topical therapy by irrigation postoperatively.

Quantitative evaluation of objective outcomes is presented in Table 3. Globally, our quantitative evaluation showed significant improvement in SNOT-22 (mean of 50.2 in pre to 29.6 in post; *p* = 0.006) and in Lund–Kennedy (mean of 8.8 in pre- to 5.1 in post-; *p* = 0.01) scores in our study population. Corroborating subjective data, patients without satisfactory subjective improvement showed no significant improvement in SNOT-22 and Lund–Kennedy scores, unlike the rest of our study population.

**Table 3:** Quantitative assessment of objective outcomes.

Data		Groups			
		With satisfactory improvement	Without satisfactory improvement	Total	*t* test *p*-value
					With vs. without improvement
Patients, total	*n* (%)	13 (81.3)	3 (18.7)	16 (100)	

SNOT-22 pre	Mean (SD)	47.7 (19.6)	61.0 (16.4)	50.2 (19.3)	0.31
SNOT-22 post	Mean (SD)	25.3 (19.3)	48.0 (16.5)	29.6 (20.4)	0.13
Difference SNOT-22	Mean (SD)	22.4 (23.4)	13.0 (4.4)	20.6 (21.6)	0.19
Pre vs. post *t* test *p*-value		0.007^a^	0.39	0.006^a^	

Lund–Kennedy pre	Mean (SD)	8.5 (3.5)	10.3 (1.5)	8.8 (3.3)	0.19
Lund–Kennedy post	Mean (SD)	4.2 (4.3)	9.3 (1.2)	5.1 (4.4)	0.002^a^
Difference Lund–Kennedy	Mean (SD)	4.3 (4.0)	1.0 (1.0)	3.7 (3.8)	0.02^a^
Pre vs. post *t* test *p*-value		0.009^a^	0.42	0.01^a^	

*N*, number; %, percentage; SD, standard deviation.

a Statistical significance.

Qualitative evaluation of objective outcomes is demonstrated in Table 4; this assessment showed that no patient exhibited poorer SNOT-22 scores, 4 (25%) showed no improvement, and 12 (75%) improved. Of the twelve patients with improvement, one of them was considered as achieving total improvement (6.3%). As to Lund–Kennedy scores, no patient suffered worsening, 4 (25%) showed no improvement, and 12 (75%) improved. Of these 12 patients, 5 (31.3%) achieved full recovery in their endoscopic score.

**Table 4:** Qualitative assessment of objective outcomes.

Patient	Gender	Age	Diagnostic	SNOT-22 pre	SNOT-22 post	Post-pre difference	Objective improvement SNOT-22	Lund–Kennedy pre	Lund–Kennedy post	Post-pre difference	Objective improvement Lund–Kennedy
1	F	52	CRSwNP	79	64	−15	Improvement	10	10	0	Without improvement
2	F	61	CRSwNP	61	12	−49	Improvement	10	7	−3	Improvement
3	M	50	CRSwNP	75	0	−75	Total improvement	9	0	−9	Total improvement
4	M	60	CRSwNP	27	15	−12	Improvement	10	0	−10	Total improvement
5	F	29	CRSwNP	67	17	−50	Improvement	10	9	−1	Improvement
6	F	56	CRSw/oNP	52	34	−18	Improvement	5	0	−5	Total improvement
7	F	59	CRSwNP	6	8	2	Without improvement	5	0	−5	Total improvement
8	F	37	CRSw/oNP	65	40	−25	Improvement	11	0	−11	Total improvement
9	M	47	CRSwNP	27	11	−16	Improvement	6	6	0	Without improvement
10	M	64	CRSwNP	47	31	−16	Improvement	12	10	−2	Improvement
11	F	58	CRSwNP	33	17	−16	Improvement	12	10	−2	Improvement
12	F	53	CRSwNP	54	61	7	Without improvement	10	10	0	Without improvement
13	F	47	CRSw/oNP	51	39	−12	Improvement	0	0	0	Without improvement
14	F	62	CRSw/oNP	51	49	−2	Without improvement	9	8	−1	Improvement
15	F	40	CRSwNP	58	58	0	Without improvement	10	8	−2	Improvement
16	F	39	CRSw/oNP	44	17	−27	Improvement	12	4	−8	Improvement

SNOT-22, Sino-Nasal Outcome Test-22; F, female; M, male; CRSwNP, chronic rhinosinusitis with polyps; CRSw/oNP, chronic rhinosinusitis without polyps.

There were no predictive factors of subjective therapeutic success in our study sample. However, patients with satisfactory subjective improvement had significantly greater reduction and lower final values for their Lund–Kennedy score (Table 3).

## Discussion

In the evaluation of difficult-to-treat patients, that is, those who had already undergone endoscopic sinonasal surgery without adequate clinical control with topical nasal corticosteroids in the form of spray and up to two cycles of oral antibiotics and/or corticosteroids in the preceding year, the present study demonstrated efficacy of high-volume nasal irrigation with a solution of budesonide, with 81.3% of success in clinical management. Although this design does not allow for a high-level of evidence, we must consider that the study achieved a high success rate in patients who had previously failed treatment with topical nasal corticosteroids in the form of spray. Considering only objective criteria, topical therapy by irrigation was able to significantly reduce SNOT-22 and Lund–Kennedy scores, resulting in 75% improvement in objective parameters.

Unfortunately, there is little evidence in the literature regarding the use of nasal irrigation with corticosteroids, because this is a non-standardized therapeutic modality (off-label). The early studies involved low-volume irrigation^19^, ^20^, ^21^; however, low-volume irrigation methods cannot reach paranasal sinuses,^12^ and are clearly inferior to high-volume irrigation,^15^ as demonstrated in the present study. The only well-designed study (a randomized controlled clinical trial – level 1b) using high-volume irrigation showed no difference among groups of irrigation with saline, nasal irrigation by spray with budesonide, and high-volume irrigation with budesonide in patients with Samter's triad soon after endoscopic sinonasal surgery.^22^ However, in this study patients who required systemic corticosteroids in the post-operative period were excluded, and this may have selected a more neutrophilic phenotype, which would not benefit so much with nasal irrigation with corticosteroids.^10^

The first pilot study with high-volume irrigation with budesonide only was published in 2009 by Steinke et al., and reported that 6 (75%) of eight patients studied showed significant improvement of sinonasal symptoms in a visual analog scale and in Lund–MacKay scores.^16^ Another retrospective study conducted by Jang et al. compared patients in periods when they used versus did not use high-volume irrigation with budesonide after endoscopic sinonasal surgery; the study showed that 55% of 60 patients had lower SNOT-20 scores, and 56% of 60 patients had lower Lund–Kennedy scores while using topical therapy by irrigation. The SNOT-20 scores measured during topical therapy by irrigation were significantly lower than without topical therapy. This was not the case with Lund–Kennedy scores.^23^ The largest study was conducted by Snidvongs et al.; these authors prospectively recruited 111 patients, also soon after their endoscopic sinus surgery, and introduced high-volume irrigation as topical therapy with significant improvement in Likert symptoms’ score and in SNOT-22 and endoscopic scores. Moreover, an adequate control in 94.6% of patients was obtained after three months, with six patients (5.4%) requiring oral corticosteroids, and only four requiring a new surgical treatment subsequently.^10^ Our study showed 75% of patients with improvement of SNOT-22 scores, similar to the study of Steinke et al.^16^ and with more success than Jang et al. study^23^; this also occurred with respect to improvement rates of Lund–Kennedy scores (75% vs. 56%). SNOT-22 scores exhibited a significant reduction in this study, as well as in Snidvongs et al.^10^ and Jang et al.^23^ studies. Lund–Kennedy scores also fell significantly in this study, similar to Snidvongs et al.^10^ study, but this was not evident in the study by Jang et al.^23^ The clinical control rate in this study was 81.3%, a figure lower than the percentage of 94.6% obtained by Snidvongs et al.^10^

It is noteworthy that the variability of results among studies could be due to the heterogeneity of the patients included. The profile of patients recruited in our study was that of difficult-to-control CRS, which, in theory, would encompass a worse prognostic group, in comparison with those in the other studies. Moreover, despite the fact that all patients in this study had been previously operated, the topical therapy by irrigation was not started in the immediate post-operative period, as it was in the study of Snidvongs et al.^10^ This lengthy interval between surgery and the onset of nasal irrigation therapy could allow a worse endoscopic appearance to develop, including blockage of sinuses by inflammatory tissue, which would not allow for a proper penetration of topical therapy by irrigation into nasal sinuses,^24^ and contribute to the lower success rate in this study. Furthermore, in the study of Snidvongs et al., with early postoperative patients, there is no way to dissociate success rates of topical therapy versus surgery itself.^10^

The corticosteroid dose is another variable that should be considered: the studies cited used 1 mg/day of budesonide or betamethasone, while in the present study our patients used budesonide 500 μg/day. We choose a lower dose because this is closer to the usual dose of budesonide nasal spray for CRS, which is 400 μg/day. But this option may have also contributed to a lower success rate of this study, when compared to that of Snidvongs et al.^10^ On the other hand, the dose we chose leads to another consideration: all patients included in this study did not achieve clinical control when using 400 μg/day of budesonide nasal spray; however, with a small increase in the amount of corticosteroids (500 μg/day) and a change in the form of administration (high-volume nasal irrigation), 81.3% of these patients did achieve clinical control. This finding can corroborate the concept that high-volume nasal irrigations with budesonide solution not only have the advantage of a drug distribution directly to paranasal sinuses, but also bring the benefit mechanical washings with saline.^24^ Thanks to the quality and quantity of aggregated evidence, irrigations with saline are a possible recommendation for use in CRS patients, while high-volume irrigation as topical therapy with corticosteroids is still considered only a therapeutic option for CSR patients.^11^

## Conclusion

High-volume irrigation with corticosteroids as topical therapy is a good option in the management of patients with difficult-to treat chronic rhinosinusitis, significantly improving their objective and subjective parameters. Both SNOT-22 and Lund–Kennedy scores improved in 75% of cases, with satisfactory subjective control in 81.3% of these patients.

## Conflicts of interest

The authors declare no conflicts of interest.

## References

[1] Fokkens W.J., Lund V.J., Mullol J., Bachert C., Alobid I., Baroody F. (2012). European position paper on rhinosinusitis and nasal polyps 2012. Rhinol Suppl.

[2] Timperley D., Schlosser R.J., Harvey R.J. (2010). Chronic rhinosinusitis: an education and treatment model. Otolaryngol Head Neck Surg.

[3] Glicklich R., Metson R. (1995). The health impact of chronic sinusitis in patients seeking otolaryngologic care. Otolaryngol Head Neck Surg.

[4] Kosugi E.M., Chen V.G., Fonseca V.M., Cursino M.M., Mendes Neto J.A., Gregório L.C. (2011). Translation, cross-cultural adaptation and validation of SinoNasal Outcome Test (SNOT): 22 to Brazilian Portuguese. Braz J Otorhinolaryngol.

[5] Mascarenhas J.G., Fonseca V.M., Chen V.G., Itamoto C.H., Silva C.A., Gregório L.C. (2013). Long-term outcomes of endoscopic sinus surgery for chronic rhinosinusitis with and without nasal polyps. Braz J Otorhinolaryngol.

[6] Pilan R.R., Pinna F.R., Bezerra T.F., Mori R.L., Padua F.G., Bento R.F. (2012). Prevalence of chronic rhinosinusitis in São Paulo. Rhinology.

[7] Sylvester D.C., Carr S., Nix P. (2013). Maximal medical therapy for chronic rhinosinusitis: a survey of otolaryngology consultants in the United Kingdom. Int Forum Allergy Rhinol.

[8] Young L.C., Stow N.W., Zhou L., Douglas R.G. (2012). Efficacy of medical therapy in treatment of chronic rhinosinusitis. Allergy Rhinol (Providence).

[9] Lund V.J. (2005). Maximal medical therapy for chronic rhinosinusitis. Otolaryngol Clin North Am.

[10] Snidvongs K., Pratt E., Chin D., Sacks R., Earls P., Harvey R.J. (2012). Corticosteroid nasal irrigations after endoscopic sinus surgery in the management of chronic rhinosinusitis. Int Forum Allergy Rhinol.

[11] Rudmik L., Hoy M., Schlosser R.J., Harvey R.J., Welch K.C., Lund V. (2013). Topical therapies in the management of chronic rhinosinusitis – an evidence-based review with recommendations. Int Forum Allergy Rhinol.

[12] Snidvongs K., Chaowanapanja P., Aeumjaturapat S., Chusakul S., Praweswararat P. (2008). Does nasal irrigation enter paranasal sinuses in chronic rhinosinusitis?. Am J Rhinol.

[13] Georgalas C., Cornet M., Adriaensen G., Reinartz S., Holland C., Prokopakis E. (2014). Evidence-based surgery for chronic rhinosinusitis with and without nasal polyps. Curr Allergy Asthma Rep.

[14] Wormald P-J., Cain T., Oates L., Hawke L., Wong I. (2004). A comparative study of three methods of nasal irrigation. Laryngoscope.

[15] Abadie W.M., McMains K.C., Weitzel E.K. (2011). Irrigation penetration of nasal delivery systems: a cadaver study. Int Forum Allergy Rhinol.

[16] Steinke J.W., Payne S.C., Tessier M.E., Borish L.O., Han J.K., Borish L.C. (2009). Pilot study of budesonide inhalant suspension irrigations for chronic eosinophilic sinusitis. J Allergy Clin Immunol.

[17] Lund V.J., Kennedy D.W. (1997). Staging for rhinosinusitis. Otolaryngol Neck Surg.

[18] Gregório L.L., Andrade J.S., Caparroz F.A., Saraceni Neto P., Kosugi E.M. (2015). Influence of age and gender in the normal values of Sino Nasal Outcome Test-22. Clin Otolaryngol.

[19] DelGaudio J.M., Wise S.K. (2006). Topical steroid drops for the treatment of sinus ostia stenosis in the postoperative period. Am J Rhinol.

[20] Sachanandani N.S., Piccirillo J.F., Kramper M.A., Thawley S.E., Vlahiotis A. (2009). The effect of nasally administered budesonide respules on adrenal cortex function in patients with chronic rhinosinusitis. Arch Otolaryngol Head Neck Surg.

[21] Kanowitz S.J., Batra P.S., Citardi M.J. (2008). Topical budesonide via mucosal atomization device in refractory postoperative chronic rhinosinusitis. Otolaryngol Head Neck Surg.

[22] Rotemberg B., Zhang I., Arra I., Payton K. (2011). Postoperative care for Samter's triad patients undergoing endoscopic sinus surgery: a double-blinded randomized controlled trial. Laryngoscope.

[23] Jang D.W., Lachanas V.A., Segel J., Kountakis S.E. (2013). Budesonide nasal irrigations in the postoperative management of chronic rhinosinusitis. Int Forum Allergy Rhinol.

[24] Harvey R.J., Debnath N., Srubiski A., Bleier B., Schlosser R.J. (2009). Fluid residuals and drug exposure in nasal irrigation. Otolaryngol Head Neck Surg.

